# An Innovative Approach to Navigating Microaggressions in Medical Education Settings

**DOI:** 10.1097/ACM.0000000000005946

**Published:** 2024-12-18

**Authors:** Nariell Morrison, Olutunmise Ashaye, Simisola Onanuga, Hannah Okechukwu, Kinan Wihba, Chioma Izzi-Engbeaya, Amir H. Sam

**Affiliations:** **N. Morrison** is a physician and clinical education fellow (equality, diversity, and inclusivity), Imperial College School of Medicine, Imperial College London, London, United Kingdom, and honorary clinical research fellow, Warwick Medical School, University of Warwick, Coventry, United Kingdom; ORCID: https://orcid.org/0000-0001-6961-0032.; **O. Ashaye** is a physician and former clinical education fellow (equality, diversity, and inclusivity), Imperial College School of Medicine, Imperial College London, London, United Kingdom.; **S. Onanuga** is a medical student, Imperial College School of Medicine, Imperial College London, London, United Kingdom.; **H. Okechukwu** is a medical student, Imperial College School of Medicine, Imperial College London, London, United Kingdom.; **K. Wihba** is a medical student, Imperial College School of Medicine, Imperial College London, London, United Kingdom.; **C. Izzi-Engbeaya** is director of equality, diversity, and inclusivity and pathway director of the pharmacology intercalated BSc, Imperial College School of Medicine, Imperial College London, and consultant endocrinologist, Imperial College Healthcare NHS Trust, London, United Kingdom; ORCID: https://orcid.org/0000-0001-7599-0166.; **A.H. Sam** is professor and head, Imperial College School of Medicine, Imperial College London, and consultant physician and endocrinologist, Imperial College Healthcare NHS Trust, London, United Kingdom; ORCID: https://orcid.org/0000-0002-9599-9069.

## Abstract

**Problem:**

Microaggressions negatively affect the experiences of medical students, especially those from minoritized groups, indicating the need for heightened awareness and open dialogue. The increasing recognition of the potential harm caused by such behaviors has led to a call for educational strategies that enable medical students to identify and address microaggressions effectively. This report details an innovative approach designed to navigate the complexities of microaggressions within medical education settings.

**Approach:**

In December 2023, 2 senior medical educators facilitated an in-person lecture, which consisted of short videos cocreated with students, interactive online surveys, and a presentation. The lecture aimed to enable third-year medical students at Imperial College School of Medicine to describe and recognize microaggressions and other forms of inappropriate behavior, understand the impact of microaggressions in medical education settings, develop problem-solving skills to challenge inappropriate behavior, and differentiate the informal and formal mechanisms to raise concerns.

**Outcomes:**

The final data set consisted of 183 participants. Participants reported increases in confidence in identifying microaggressions (median [IQR], 4.00 [3.00–4.00] before vs 4.00 [4.00–5.00] after intervention; *P* < .001), understanding their potential effect on affected individuals (median [IQR], 4.00 [3.00–5.00] before vs 5.00 [4.00–5.00] after intervention; *P* < .001), and feeling better equipped to challenge inappropriate experienced (median [IQR], 2.00 [2.00–3.00] before vs 3.00 [2.00–4.00] after intervention; *P* < .001) or witnessed (median [IQR], 3.00 [2.00–3.00] before vs 4.00 [3.00–4.00] after intervention; *P* < .001) behaviors. They also reported increases in confidence in seeking support from their peers if they experienced (median [IQR], 4.00 [3.00–5.00] before vs 4.00 [4.00–5.00] after intervention; *P* < .001) or witnessed (median [IQR], 4.00 [3.00–4.00] before vs 4.00 [3.00–5.00] after intervention; *P* < .001) microaggressions.

**Next Steps:**

Next steps include integrating small group workshops on microaggressions into curricula and adapting these interventions for other health care professionals.

## Problem

Within medical education, there is increasing concern about microaggressions and their effect on medical students.^1,2^ Microaggressions are defined as commonplace verbal, behavioral, or environmental indignities that convey hostility or negative attitudes, regardless of intention, toward targets’ identities.^3^ They manifest in various forms, including microassaults, microinsults, and microinvalidations. Microassaults are more overt and deliberate verbal or behavioral attacks that are discriminatory or demeaning. Microinsults are often unwitting comments that degrade or offend the target, and microinvalidations frequently dismiss or minimize the experiences or feelings of the target.^3^ These behaviors, despite their “micro” prefix, which emphasizes the interpersonal nature of these interactions rather than their impact, can often go unnoticed by bystanders, further complicating the issue.^3^ Moreover, within medical school and clinical environments, microaggressions may originate from various sources, including students, faculty, staff, and patients, affecting not only the targeted individuals but also those who witness them, thereby contributing to a hostile learning environment.

The impact of microaggressions is profound, potentially causing significant psychological and physiologic distress, increasing cognitive load, and activating stereotype threats among medical students, particularly those from minoritized groups.^3^ These consequences can severely impede learning and performance, adversely affecting students’ professional development and future career paths.^1^ Recent efforts toward diversifying the medical student body to better reflect the demographics of the populations they will serve have led to increased representation of women, sexual and gender minority groups, and racial and ethnic groups historically marginalized in medicine. However, this diversification has, paradoxically, elevated the likelihood of students experiencing and witnessing microaggressions.^4^

After our institution, Imperial College London, signed up to the British Medical Association’s Racial Harassment Charter in 2020 and following concerns raised formally and informally by students, an independent external consultant identified areas for improvement after conducting focus groups and interviews with students at our medical school. Consequently, we urgently recognized the need for educational interventions to raise awareness of microaggressions and facilitate effective dialogue to address these concerns. These efforts are crucial for cultivating a sense of belonging and an inclusive learning environment for all students, essential for achieving optimal educational outcomes.

Despite attempts to address microaggressions in medical education,^5–7^ studies describing educational initiatives to tackle these issues within U.K. medical schools have been scarce. In response, we developed and implemented an innovative, video-based interactive lecture as part of our commitment to embed equality, diversity, and inclusivity (EDI) within the learning environment. This lecture, Navigating Inappropriate Behavior in Medicine, aimed to enhance medical students’ understanding of microaggressions, thus empowering them with the knowledge and strategies to handle such incidents. In this Innovation Report, we detail the design of a video-based curriculum to effectively address microaggressions and evaluate its potential to foster a more inclusive and supportive medical student learning environment. To our knowledge, we are the first U.K. medical school to use videos cocreated by faculty and students in microaggressions training.

## Approach

### Setting

We incorporated an in-person EDI lecture, entitled Navigating Inappropriate Behavior in Medicine, into the curriculum for third-year medical students at Imperial College London. Although EDI is embedded throughout the medical curriculum, this lecture marks the students’ first specific exposure to learning about microaggressions and applying their knowledge practically. With this innovation, we aimed to initiate a dialogue on microaggressions and inappropriate behavior within the medical field, offering insights into the potential impact of such behaviors and equipping students with strategies for managing these situations should they witness or experience them. This study received ethical approval from the Imperial College London Education Ethics Review Process. The lecture, including the videos, was presented to students as a mandatory component of the standard teaching provision; however, participation in this study was entirely voluntary. All participants provided informed consent before completing the questionnaires.

### Curriculum development

**Figure 1:**
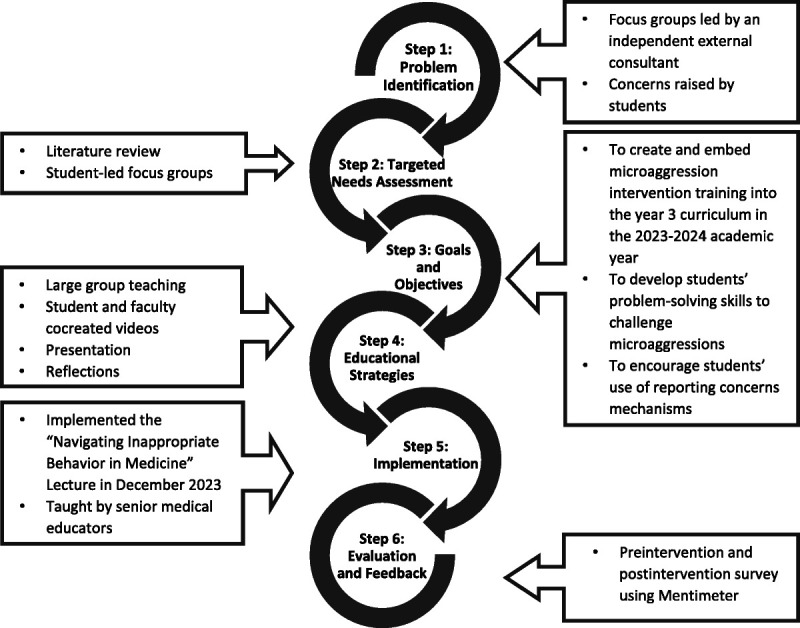
Steps taken to create the video-based curriculum to effectively address microaggressions in the medical education setting, based on the 6-step approach of Thomas et al.^8^

Figure 1 is a flow diagram of the steps we took to create our curriculum. We applied the 6-step approach of Thomas et al^8^ to curriculum development, informed by Bandura’s social learning theory,^9^ to construct our conceptual framework. Using this framework, we systematically created our educational intervention, using observational learning to address microaggressions in medical education. Our targeted needs assessment included a literature review and focus group interviews with medical students at Imperial College London. Using thematic analysis, we identified 4 themes: common microaggressions, the absence of allyship and bystander support, challenges in addressing microaggressions, and barriers in reporting concerns. These themes led us, faculty and students, to collaborate and cocreate 3 short videos that explore and model different scenarios. Video-based case materials have been reported to be an engaging way of learning.^10^ One video focused on disability microaggressions, another on racial microaggressions, and another on transgender microaggressions. The source of the microaggression varied in each video, involving a senior clinician, a patient, and a peer, respectively, to highlight the diverse contexts in which such behaviors can occur. On the basis of our students’ experiences and the identified skill gaps in our needs assessment, we designed our lecture with specific goals and objectives, incorporating the cocreated videos.

We integrated short writing and reflection exercises throughout the lecture as a pivotal learning strategy, facilitated through interactive free-text surveys using the Mentimeter platform (Mentimeter, Stockholm, Sweden). Participants could articulate their thoughts and experiences during the lecture without seeing other participants’ responses. In the reflection exercises, we asked participants to describe the microaggressions they witnessed in the videos and reflect on how witnessing these microaggressions made them feel, whether these feelings surprised them or made them uncomfortable, and how this awareness would impact or change their practice. We also asked participants to consider how they might support a friend or colleague who experienced a microaggression and was upset by it.

### Curriculum implementation

In December 2023, we scheduled a 90-minute lecture led by 2 senior medical educators (C.I.-E. and A.H.S.). Participants completed a Likert scale survey before and after the intervention that assessed confidence in identifying various forms of microaggressions, understanding their potential effect on affected individuals, and actively challenging inappropriate behavior if they experienced or witnessed a microaggression. Additionally, the surveys assessed participants’ confidence in seeking support from peers and the medical school if they experienced or witnessed a microaggression. The surveys also measured participants’ confidence in reporting such instances and their belief that the medical school would take appropriate action when microaggressions are reported (see Supplemental Digital Appendix 1 at http://links.lww.com/ACADMED/B653). Responses were given on a 5-point scale, with 1 indicating strongly disagree and 5 indicating strongly agree. Using the same device, participants completed each survey anonymously on Mentimeter, and responses before and after the intervention were automatically matched using the platform’s software. Given the potentially sensitive nature of the topics discussed, we provided trigger warnings and advance notices to students. The face-to-face teaching format, which included videos, interactive surveys (Mentimeter), and a presentation, aimed to enable medical students to achieve the following outcomes: (1) describe and recognize microaggressions and other forms of inappropriate behavior; (2) understand the impact of microaggressions in medical education settings, including clinical environments; (3) develop problem-solving skills to challenge inappropriate behavior, including microaggressions; and (4) differentiate the informal and formal mechanisms to raise concerns. Table 1 summarizes the alignment of the intended learning outcomes, lecture content, and evaluation tool.

**Table 1:** Alignment of Intended Learning Outcomes, Lecture Content, and Evaluation Tool of Imperial College London’s “Navigating Inappropriate Behavior in Medicine” Lecture

Intended learning outcomes	Lecture content	Evaluation tool
Describe and recognize microaggressions and other forms of inappropriate behavior	Student and faculty cocreated short videos on microaggression theory (definition, taxonomy, and relevance to medicine)	10-Item confidence preintervention and postcurriculum intervention surveys
Understand the impact of microaggressions in medical education settings, including clinical environments	Effect of microaggressions on physical and psychological (cognitive, emotional, and behavioral) health	
Develop problem-solving skills to challenge inappropriate behavior, including microaggressions	Suggested strategies to navigate microaggressions	
Differentiate the informal and formal mechanisms to raise concerns	Imperial School of Medicine and Imperial College London reporting concerns mechanisms and student support systems	

### Statistical analysis

We analyzed data using IBM SPSS Statistics, version 29.0 (IBM Corp., Armonk, New York). We compared self-reported confidence responses to each statement in the surveys before and after the curriculum intervention using Wilcoxon signed-rank tests for matched pairs. A 2-sided *P* < .01 was considered statistically significant.

## Outcomes

Of 242 third-year medical students who participated in the lecture, 10 (4.1%) did not consent to having their anonymized data processed as part of the empirical study. In addition, we excluded 49 students (20.3%) because they left the consent question unanswered, resulting in a final sample size of 183 (75.6%).

Results demonstrated overall improvement in confidence after the curriculum intervention. The median (interquartile range [IQR]) scores before and after the curriculum intervention survey are given in Table 2. Participants reported statistically significant increases in their confidence in identifying microaggressions (median [IQR], 4.00 [3.00–4.00] before vs 4.00 [4.00–5.00] after the intervention; *P* < .001), understanding their potential effect on affected individuals (median [IQR], 4.00 [3.00–5.00] before vs 5.00 [4.00–5.00] after the intervention; *P* < .001), and feeling better equipped to actively challenge inappropriate behaviors that are experienced (median [IQR], 2.00 [2.00–3.00] before vs 3.00 [2.00–4.00] after the intervention; *P* < .001) or witnessed (median [IQR], 3.00 [2.00–3.00] before vs 4.00 [3.00–4.00] after the intervention; *P* < .001). They also reported statistically significant increases in their confidence in seeking support from their peers if they experienced (median [IQR], 4.00 [3.00–5.00] before vs 4.00 [4.00–5.00] after the intervention; *P* < .001) or witnessed (median [IQR], 4.00 [3.00–4.00] before vs 4.00 [3.00–5.00] after the intervention; *P* < .001) a microaggression. Finally, there were statistically significant increases in confidence ratings among participants in seeking support from (experiencing microaggression: median [IQR], 2.00 [1.00–3.00] before vs 3.00 [3.00–4.00] after the intervention; *P* < .001; witnessing microaggression: median [IQR], 2.00 [1.00–3.00] before vs 3.00 [3.00–4.00] after the intervention; *P* < .001) and reporting incidents to (median [IQR], 2.00 [1.00–3.00] before vs 4.00 [2.50–4.00] after the intervention; *P* < .001) the medical school as well as increased confidence in the institution taking appropriate actions following the receipt of such reports (median [IQR], 3.00 [1.00–3.00] vs 3.00 [2.00–4.00]; *P* < .001).

**Table 2:** Survey Responses of 183 Third-Year Medical Students Before and After They Participated in Imperial College London’s “Navigating Inappropriate Behavior in Medicine” Lecture

Survey statement	Before intervention, median (IQR)^a^	After intervention, median (IQR)^a^	Wilcoxon	
			*z*	*P* value
I feel confident in identifying the various forms microaggressions can take, whether verbal, nonverbal, or environmental	4.00 (3.00–4.00)	4.00 (4.00–5.00)	3.528	< .001
I feel confident in my understanding of the potential effect of microaggressions on affected individuals	4.00 (3.00–5.00)	5.00 (4.00–5.00)	4.236	< .001
I feel confident in actively challenging inappropriate behavior if I experience a microaggression	2.00 (2.00–3.00)	3.00 (2.00–4.00)	5.987	< .001
I feel confident in actively challenging inappropriate behavior if I witness a microaggression	3.00 (2.00–3.00)	4.00 (3.00–4.00)	6.964	< .001
I feel confident in seeking support from my peers if I experience a microaggression	4.00 (3.00–5.00)	4.00 (4.00–5.00)	3.945	< .001
I feel confident in seeking support from my peers if I witness a microaggression	4.00 (3.00–4.00)	4.00 (3.00–5.00)	4.501	< .001
I feel confident in seeking support from the medical school if I experience a microaggression	2.00 (1.00–3.00)	3.00 (3.00–4.00)	6.777	< .001
I feel confident in seeking support from the medical school if I witness a microaggression	2.00 (1.00–3.00)	3.00 (3.00–4.00)	6.339	< .001
I feel confident in reporting any experiences of or instances where I witnessed microaggressions to the medical school	2.00 (1.00–3.00)	4.00 (2.50–4.00)	5.324	< .001
I feel confident that the medical school will take appropriate action when instances of microaggressions are reported	3.00 (1.00–3.00)	3.00 (2.00–4.00)	4.426	< .001

^a^Responses were given on a 5-point scale, with 1 indicating strongly disagree and 5 indicating strongly agree.

## Next Steps

To our knowledge, our student and faculty cocreated video-based educational intervention is the first of its kind undertaken in a U.K. medical school. Informed by social learning theory, it builds on previous work around educating medical students and faculty on microaggressions.^1,2,4–7^ However, we acknowledge our study had some limitations. First, the outcome measures of interest relied exclusively on self-reported ratings of perceived confidence, and the postintervention measurements were taken immediately after the lecture. Consequently, our results should be interpreted with caution due to the uncertainty regarding the permanence of these changes. Future iterations could assess confidence over a longer period, providing a more accurate measure of the long-term effects of this educational intervention. Second, our study only reports findings from a single cohort at 1 medical school (i.e., the third year of a 6-year course in a large medical school with a diverse mix of students from different ethnic and socioeconomic backgrounds). The generalizability of our findings to medical students from different contexts needs to be explored in further studies. Third, we cannot determine the extent to which our results are more generalizable in terms of recognizing other forms of microaggressions or more subtle versions of those displayed. We recommend conducting follow-up studies in which students identify de novo examples to evaluate these issues. Despite these limitations, we hope this report will inspire further efforts to equip medical students with the necessary knowledge and skills to effectively respond to microaggressions they may witness or experience.

By fostering an open learning environment to address microaggressions and other forms of inappropriate behavior early in medical careers, medical schools could make an important contribution to reducing the incidence of such behaviors, thus improving students’ well-being and sense of belonging. Although our intervention reached only a fraction of the student body, it sparked broader discussions within the student community. As a result, we have planned the integration of similar lectures, as well as small group workshops, on microaggressions into the curriculum for other cohorts. By sharing our experiences and results, we hope to encourage the adoption of this innovative approach by other medical schools.

Next steps include adapting our novel lecture to teach medical educators, physicians, and other health care professionals (e.g., nursing, pharmacy, and/or physiotherapy students) to open dialogues and empower students and educators to change the institutional climates of medical schools and health systems. Additional videos could be created that explore other forms of microaggressions or increase the complexities depicted, such as combining scenarios of acutely unwell patients with microaggressions. This approach would be valuable as we further embed EDI into medical education.

Additionally, our work provides future research opportunities, such as qualitative exploration using interviews or focus groups after the intervention, to assess the longer-term retention of the learning material and the application of learned skills in real-world settings, whether within the medical school or clinical environments. Furthermore, research comparing the effectiveness of various teaching modalities (e.g., workshops, lectures, immersive simulations) could yield insights into the most effective methods for teaching about microaggressions. We have demonstrated that the use of videos (cocreated with students) in large group teaching is an effective method of improving the confidence of medical students to recognize and navigate microaggressions. Further work is required to assess the long-term impact of this educational intervention.
